# Applications of Near Infrared Photoacoustic Spectroscopy for Analysis of Human Respiration: A Review

**DOI:** 10.3390/molecules25071728

**Published:** 2020-04-09

**Authors:** Dan C. Dumitras, Mioara Petrus, Ana-Maria Bratu, Cristina Popa

**Affiliations:** 1 University “Politehnica” of Bucharest, Physics Department, Faculty of Applied Sciences, University “Politehnica” of Bucharest, 313 Splaiul Independentei, 060042 Bucharest, Romania; 2 National Institute for Laser, Plasma and Radiation Physics, Laser Department, 409 Atomistilor St., PO Box MG 36, 077125 Magurele, Romania; mioara.petrus@inflpr.ro (M.P.); ana.magureanu@inflpr.ro (A.-M.B.); cristina.achim@inflpr.ro (C.P.)

**Keywords:** photoacoustic spectroscopy, breath analysis, biomarkers

## Abstract

In this review, applications of near-infrared photoacoustic spectroscopy are presented as an opportunity to evaluate human respiration because the measurement of breath is fast, intact and simple to implement. Recently, analytical methods for measuring biomarkers in exhaled air have been extensively developed. With laser-based photoacoustic spectroscopy, volatile organic compounds can be identified with high sensitivity, at a high rate, and with very good selectivity. The literature review has shown the applicability of near-infrared photoacoustic spectroscopy to one of the problems of the real world, i.e., human health. In addition, the review will consider and explore different breath sampling methods for human respiration analysis.

## 1. Introduction

Even from the beginning breath analysis research was performed with the aim of disease diagnosis. Analysis of breath from humans has a long history, dating back to the time of Hippocrates (460–370 BC), when the ancient Greek physicians realized that the aroma of human breath could provide information on health conditions [1,2,3,4,5,6]. Later in 1782, Lavoisier was the first who studied the breath CO_2_ of guinea pigs and showed that the gas is a product of combustion in the body [5,6]. Later, many studies have reported the significance of analyzing volatile organic compounds (VOCs) in human breath [7,8,9]. In 1931 Harger invented the ‘drunkometer’ for testing breath-alcohol concentration and in 1938 it became commercially available. Current breath analysis started to show great potential with a discovery made by Pauling et al. in 1971 [10]. They used gas chromatography (GC) to detect more than 200 VOCs in human breath. The science of volatile metabolomics has expanded and many studies have been carried aiming to characterize these VOCs. Reported results from Phillips estimated 1,259 compounds in normal subjects in 1997 [11], and over 3,000 compounds in 1999 [12].

In the recent years, breath analysis research focused on the identification of some volatiles as specific biomarkers for the management of patients and, particularly with regard to early diagnosis. Humans breath contains a mixture of nitrogen, oxygen, carbon dioxide, inert gases, water vapor and thousands of VOCs trace and inorganic molecules [13]. The composition of breath contains exogenous compounds that originate from environmental exposures, and endogenous compounds that are produced by biological processes like oxidative stress (OS) and inflammation in the human body [14]. The complex matrix of breath varies from each person both quantitatively and qualitatively. A number of compounds are considered biomarkers that generates information of great importance on human status [15,16,17,18,19,20]. The development of different methods allow the detection of volatiles present in the breath at low concentrations. The most commonly analytical tools for detection of breath molecules include proton transfer reaction mass spectrometry (PTR-MS) [21], selected ion flow tube mass spectrometry (SIFT-MS) [22], gas chromatography-mass spectrometry (GC-MS) [23], e-noses [24], and laser-based sensors [25,26]. Today, laser spectroscopic detection techniques like tunable diode laser absorption spectroscopy (TDLAS), cavity-enhanced absorption spectroscopy (CEAS), integrated cavity output spectroscopy (ICOS), cavity ringdown spectroscopy (CRDS), laser photoacoustic spectroscopy (LPAS), and quartz-enhanced photoacoustic spectroscopy (QEPAS) have reached a mature states being used in many research directions [27]. Both (PTR-MS) and (SIFT-MS) provide easy, fast, and direct analysis as they do not rely on time-consuming sample separation like GC/MS. PTR-MS is most appropriate for studies of gas mixtures with well-known composition and when working with dilute samples. SIFT-MS is better for complex mixtures of unknown composition but has low detection selectivity and requires frequent calibrations [28,29]. Both are used in various from both natural and anthropogenic atmospheric chemistry [30,31], plant studies [32], food science [33], and medical applications like breath-analysis [34,35,36]. GC-MS can analyze multiple compounds simultaneously with detection-sensitivity of ppb (parts-per-billion) to ppt (parts-per-trillion) but requires complicated procedures for sample collection and pre-concentration and also has high instrument costs [37,38,39]. GC-MS instruments are favorite for analysis in the fields of environmental science, forensics, health care, medical and biological research, health and safety, the flavor and fragrances industry, food safety, packaging, and many others. E-noses contain multiple sensors that interact with complex gaseous mixtures and provide a signal pattern but unknown compounds cannot be detected. These technologies offer noninvasive accurate diagnoses and have been applied in the medical field for analyzing volatile biomarker metabolites in the human breath [40].

The detection and quantification of trace gases from human breath is of great interest medical monitoring and diagnostics and require gas sensors characterized by high sensitivity and selectivity (to avoid interference from other potential interfering species), multi-component capability, real time measurements, large dynamic range, *in situ* measurements, ease to use. Real-time analysis of breath can be achieved using LPAS, which is sufficiently sensitive and rapid to allow the simultaneous analyses of several trace gas metabolites in single breath exhalations. Over the years, photoacoustic spectroscopy (PAS) has been demonstrated to be capable of sensing trace gases with the quality and facility required for biological and medical applications. This technique has limits ranging from the part-per-million (ppm, μmol/mol) to less than part-per-billion (ppb, nmol/mol) and it is characterized by long-term stability, high sensitivity and selectivity, detection of one or several gases, real-time analysis and maintenance of free conditions [25,26]. Later experiments introduced the photoacoustic (PA) effect into the field of trace gas detection with environmental, biological, and medical applications. The capability of LPAS has been increased with significant improvements in light sources, modulators and PA signal transducers having successful applications to atmospheric environmental monitoring, chemical analysis, industrial process control, medical diagnostics and applications of life science, etc. [25,26,41,42,43,44,45,46,47,48,49,50,51,52].

In this review we report recent developments on LPAS in human breath analysis. After a preliminary section, the paper is divided into two sections. In the first section, we describe the fundamentals of LPAS for trace gas detection: the theoretical background, light sources, photoacoustic cell, noises and limiting factors. In the second section, we present the results in the field of human health reported by different groups by analyzing breath biomarkers using LPAS.

## 2. Laser Photoacoustic Spectroscopy

Laser photoacoustic detection techniques as a tool for trace gas analysis can be used to monitor many different samples simultaneously, while offering many important attributes like high sensitivity and selectivity, large dynamic range, high accuracy and precision, good temporal resolution, versatility, reliability, robustness and is ease of use [53,54]. The techniques have undergone continuous development and can be applied today in almost all disciplines of science and technology [41,42,43,44,45,46,47,48,49,50,51,52,53,54,55,56].

LPAS is based on the photoacoustic (PA) effect that occurs at the interaction of light and matter and sound is generated. This phenomenon was discovered in 1880 by Alexander Graham Bell [57], who while studying wireless communication discovered that optically absorbing solid substances emit a sound when are illuminated by a modulated light. One year later Bell [58], Tyndall [59], Röntgen [60] and Preece [61] demonstrated that the photoacoustic effect appear not only in solids but also in liquids and gases and the sound was stronger when the substance was placed in a sample cell named photophone and later spectrophone. The interest in the photoacoustics has decreased for decades until the appearance of sensitive microphone.

**Figure 1:**
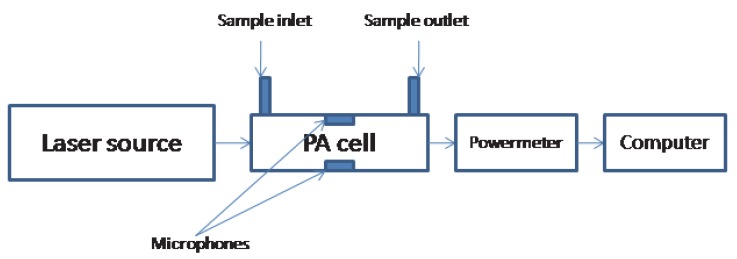
Schematic of photoacoustic spectroscopy.

In PAS, as illustrated in Figure 1, the absorbing medium (e.g., a gas sample) enclosed in a specially designed PAS cell absorbs laser radiation at a selected frequency and the photon energy absorbed by the gas is translated into gas pressure variations through non-radiative relaxation processes which give rise to acoustic waves detected by sensitive microphones. [25,26,53].

### 2.1. Theoretical Background

Laser spectroscopy comprise three techniques according to measurement of different physical quantities: the absorption method and the cavity ringdown spectroscopy (intensity), the radiative method (fluorescence), the photothermal (calorimetric) method (pressure, temperature) [25,26]. PA spectroscopy is a technique that measures indirect absorption condition, absorption of light is detected by the accompanying sound effect. 

**Figure 2:**
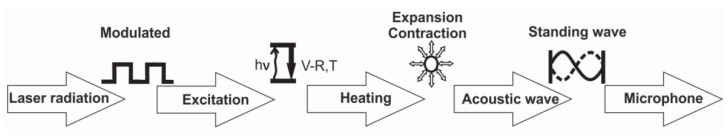
Schematic of the physical processes occurring in PAS [25].

The PA effect in gases can be divided into five main steps (see Figure 2) [25]:

(1) Modulation of the laser radiation (either in amplitude or frequency) at a wavelength that overlaps with a spectral feature of the target species;
(2) Excitation o of the target molecule by absorption of the incident laser radiation;
(3) Energy exchange processes: the energy which is absorbed is almost completely converted to the kinetic energy of the gas molecules, the kinetic energy and then converted into periodic local heating at the modulation frequency;
(4) Expansion and contraction of the gas in a closed volume that give rise to pressure variation which is an acoustic wave;
(5) Detection of the resulting acoustic waves with microphones.

In spectroscopic trace gas detection the most important optical process is based on the extinction of radiation by molecular absorption. Identification of trace gases and determination of their concentration depends of the absorption coefficients of each molecule, that are typically on the order of 1 cm^−1^ (one wave number). In a gas mixture the absorption of trace gas molecules may be monitored by detecting the attenuation of the laser beam over a fixed absorption path length *L*.

According to the Beer-Lambert law, the transmitted laser power in the absence of saturation is given by:

(1) $$P\left(L\right)=P\left(0\right)\exp\left(-\alpha_{p}L\right)=P\left(0\right)\exp\left(-\alpha \mathrm{cL}\right),$$

where *P*(0) is the laser powers before the absorption cell, *P*(L) is the laser powers after the absorption cell, *α_p_* (cm^−1^) is the absorption coefficient at a given pressure of the gas at a specific laser wavelength. Here *α_p_* = *αc,* *α*(cm^−1^ atm^−1^) is the gas absorption coefficient (the absorption coefficient normalized to unit concentration), and c (atm) is the trace gas concentration. At 1013 mbar and 20 °C, *α_p_* = *N*_tot_*σ*, where *σ* (cm^2^) is the absorption cross section per molecule and *N*_tot_ = 2.5 × 1019 molecules cm^−3^ is the number of absorbing molecules per cubic centimeters.

This results in:

(2) $$c=\frac{-1}{\alpha L}\ln\frac{P\left(L\right)}{P\left(0\right)}=\frac{-1}{\alpha L}\ln\left(1-\frac{\Delta P}{P\left(0\right)}\right)≃\frac{1}{\alpha L}\frac{\Delta P}{P\left(0\right)},$$

This relation is valid for Δ*P*/*P*(0) << 1 (i.e., an optically thin sample), where Δ*P* = *P*(0) − *P*(L). The detection limit is given by the smallest relative change Δ*P*_min_/*P*(0) for a given *L*, that can be measured in the transmitted signal. The desired signal for diluted mixtures and modest lengths of the absorption path is shown by the small difference between two large values so that are necessary high quantitative accuracies in signal intensities. Frequency modulation and harmonic detection are used in the most sensitive method where for atmospherically broadened lines the sensitivity depends on the linewidth.

The PA voltage signal at a specific operating frequency (when the resonance contributions are included) is given by multiplying the pressure response ($p=C\left(\omega =\omega_{0}\right)\alpha_{p}P_{L}$) by the microphone responsivity (*V* = *pS*_M_ and *α_p_* = *αc*) where *p* (N/m^2^ = Pa) is the pressure response of the cell, *α_p_* (cm^−1^) is the absorption coefficient at a given pressure of the gas at the laser wavelength, and *P*_L_ (W) is the laser power. Here, the angular frequency is *ω*_0_ = 2*πf*_0_, where *f*_0_ is the resonance frequency; for a longitudinal resonant cell, the first resonance frequency is *v_s_*/2*L*, so that *ω*_0_ = *πv_s_*/*L*. The quantity *C* (Pa cm/W) is the cell constant usually determined by calibration measurements, where one single absorbing substance with known absorption spectrum is investigated.

In that case:

(3) $$V=\alpha {\mathrm{CS}}_{M}P_{L}c$$

where: *V* (V) is the voltage measured at peak-to-peak value; *α* (cm^−1^ atm^−1^) represents the gas absorption coefficient at a given wavelength; *C* (Pa cmW^−1^) is the cell constant; *S*_M_ (VPa^−1^) is the microphone responsivity; *P*_L_ (W) is the unchopped laser beam value; and *c* (atm) represents the trace gas concentration (usually given in units of per cent, ppmV, ppbV or pptV (parts-per-trillion by volume)).

According to this equation the PA signal is linearly dependent on the absorption coefficient, cell constant, microphone responsivity, incident laser power, and absorbent trace gas concentration, which means that this technique is a “zero-baseline” approach, since no signal will be generated if the target molecules are not present. The peak-to-peak value of the signal is obtained multiplying by 2 the rms voltage amplitude measured by the lock-in amplifier.

Another parameter is used to characterize the PA cell:

(4) $$R={\mathrm{CS}}_{M},$$

where *R* (V cm/W) is the (voltage) responsivity of the PA cell or the calibration constant. The cell constant *C* is multiplied by the responsivity of the microphone given in V/Pa units. In this way:

(5) $$V=\alpha {\mathrm{RP}}_{L}c$$

The minimum detectable concentration *c* = *c*_min_ of a target trace gas can be recorded based on the minimum measurable voltage signal *V*= *V*_min_ achieved when the signal to ratio is unitary (SNR = 1):

(6) $$c_{\min}=\frac{V_{\min}}{\alpha P_{L}R}$$

In contrast to other techniques based on absorption spectroscopy, the response of the acoustic detector is independent of the electromagnetic radiation wavelength as long as the absorption coefficient is fixed. Extremely low detection limits on the order of *α_min_* = *αc_min_* ≅ 10^−8^ cm^−1^ for 1 W incident laser power have been achieved. Such sensitivity makes possible the detection of many trace constituents in the sub-ppbV range.

### 2.2. Laser Sources

Due to its high sensitivity, LPAS allows single breath collection from a small sampling volume (a few 100 mL) with no pre-concentration steps needed. Within LPAS a high sensitivity can be achieved with high-power infrared lasers. In addition, a wide-tunability is required to selectively detect gases in complex gas mixtures and to operate at the optimal wavelength region to minimize spectroscopic interference from other gases such as water. Such widely tunable high power lasers are available, for example CO and CO_2_ lasers, optical parametric oscillators (OPOs), or external cavity quantum cascade lasers (EC-QCLs) [25,26].

The main characteristics of laser determine the unique properties of LPAS. The spectral overlapping of the laser emission with the absorption bands of the trace gas molecules determine the kind and number of detectable substances. Spectral resolution of the laser, the accessible wavelength range, tunability are essentially for this kind of detection [62]. LPAS sensitivity, given by the minimum detectable concentration, is supported mostly by lasers in the IR region were molecules of interest exhibit characteristic absorption lines. The suitable laser sources also control the selectivity (tuning range), practicability (ease of use, size, cost, and reliability) and time resolution (the time needed for laser tuning and the gas exchange within the cell) of LPAS.

CO and CO_2_ laser present many advantages for trace gas monitoring system based on LPAS. CO_2_ lasers are line tunable lasers that cover the infrared 9–11 µm wavelength region with a laser line spacing of 0.5–2 cm^−1^. CO_2_ lasers offers continuous tunability in the wavelength range of 9 to 11 µm which extends to 12 µm when different CO_2_ isotopes are used. CO_2_ can be gradually tuned when operates in cw and is the best possible source for the detection and quantification of molecular trace gases with detection sensitivities ranging from ppmv and ppbv, to even pptv levels. The use of line-tunable infrared lasers make possible to investigate breath analysis and to advance from invasive, time-consuming procedures to non-invasive testing to obtain strategic information for clinical diagnostics. These lasers are simply to operate and can provide high laser powers from a relatively small gas discharge tube [25,26].

CO lasers are line tunable with a line spacing between 0.5–1 cm^−1^ emitting radiation in the infrared wavelength region Δʋ_1_ (5.0–7.6 µm wavelength region) and Δʋ_2_ (2.5–3.8 µm). This kind of lasers are generally less powerful, but their operation can be improved using an intracavity set-up [25,26].

PAS in infrared wavelength region has progressed through development of high power (cw and pulsed), QCLs operating at room temperature with power levels up to 1 W. QCLs operate in the mid-infrared wavelength region from 3.5 to 24 μm and now are commercially available external cavity QCL, single-mode tuning ranges, approaching 300 cm^−1^ [63,64].

A growing interest in PAS for the 2.5–5 μm region is represented by cw periodically poled lithium niobate optical parametric oscillators (PPLN-OPOs) at high power (several watts) level and narrow linewidth. OPOs are pumped by high power lasers and its outstanding features are wide-range wavelength tunability of the emitted radiation with high and stable output power levels, and an exceptional beam quality and these characteristics make OPOs very valuable for laser spectroscopy [65,66,67].

Although, for PA detection tunable laser are mostly used there are advantages of using also pulsed lasers. They have wider infrared tunability and consequently better spectral overlap with interesting molecular gases. Repond and Sigrist [68] performed experiments with a pulsed CO_2_ laser and with a pulsed OPO [69]. Pulsed lasers have the disadvantage of high peak powers (megawatt) in relation to their relatively low average energy (typically 1 W) which has to generate a relatively slow process as the gas phase photoacoustic effect. Photoacoustic studies on trace detection were performed also with other cw lasers from the visible and infrared region such as a spin flip Raman laser [70], diode lasers [71,72], step-tunable DF lasers [73], and dye lasers [74]. These are less applicable for the detection of trace gases having increased detection limits due to weak molecular absorption cross sections.

### 2.3. Photoacoustic Cell

The cavity in which the amplified and generated PA signal, initiated by absorbing molecules, was named PA cell. The main characteristics of photoacoustic cells for trace gas detection are referred to small size, simplicity, a low gas consumption or a fast response. In PAS for trace gas detection, the role of the PA cell is to amplify the generated waves from the molecular gas absorption and decline the acoustic or electric noise. These PA cells can be operated either in resonant or non-resonant mode [25,26,67].

Resonant cells are usually combined with cw lasers and non-resonant cells with pulsed lasers. In the case of non-resonant cells, modulation frequency is much lower than the first acoustic resonance frequency and the wavelength of the generated acoustic wave is larger than cell dimensions so because of this a generation of a standing acoustic wave is not possible. For resonant cells the PA signal and signal-to-noise ratio (SNR) decreased almost linearly with pressure whereas for non-resonant cells remains almost constant as pressure decreased. Attachment of the microphones to small and non-resonant cell can cause difficulties in obtaining the appropriate pressure response signal. The use of many microphones is recommended in resonant cells as sensing elements of the acoustic waves. There are varied ways to design PA cells [25,26,67].

Over time had been designed numerous types of PA cell like multipass [74,75], extracavity or intracavity [25,76] that can be cylindrical geometry, H geometry, T geometry, or Helmholtz resonator and can operate longitudinal, azimuthal, radial, or Helmholtz resonances. Comparing experimental parameters from extracavity PA cell and the intracavity PA cells it is clearly that in real PA instruments, the minimum measurable signal is higher in extracavity PA cells and much higher in intracavity PA cells (hundreds or even thousands of times) than the coherent acoustic background noise, and the best sensitivity is obtained in extracavity PA instrument, with minimum detectable absorptivity being better by one or two orders of magnitude than in intracavity arrangements [25,67].

### 2.4. Noises and Limiting Factors

The LPAS present a high sensitivity and selectivity for trace gas detection and allows on-line measurements. Noise plays an important role in all PA measurements and is of particular importance in trace detection, because the noise level limits the sensitivity [25,67,77,78]. The sensitivity-limiting factors which are encountered in LPAS can be classified into three categories [25,67]:

(a) Electrical noise, represent any random fluctuation (electronic or acoustic) which does not have a fixed phase relation with the modulation of the laser intensity.
(b) Coherent acoustic background noise, meaning a signal caused by the modulation process, but not attributable to the presence of the light beam in the PA cell.
(c) Coherent photoacoustic background signal. This signal, which is always present in the PA detector, is caused by the laser beam, it is due to laser beam heating of the windows and of the absorbates at their surfaces, and heating of the PA resonator walls by the reflected or scattered light owing to imperfections of the focusing lens, windows and inner walls of the PA resonator. This signal is in phase with, and at the same frequency as, the laser intensity modulation and therefore, it is not filtered out by the lock-in amplifier connected to the microphone. Thus, a background signal proportional to the laser power becomes the main factor that limits sensitivity.

Electrical noise can be reduced by using lock-in amplifiers and/or by using longer time averaging (the noise decreases with the square root of the averaging time) [26]. External perturbation sources may have a fixed phase relation, such as the acoustical sound caused by the mechanical chopper. This noise must be minimized in the same way as the external acoustical noise. A way to minimize the noise caused by a mechanical chopper is to find a good chopper position and to remove the objects in the neighborhood of the chopper wheel [67].

For PAS, “noise” often has a structure that is coherent with the signal from the target species, and therefore should more appropriately be treated as a background signal, not as noise. The background signal can be determined by measuring the acoustic signal in the absence of absorbers (i.e., with pure nitrogen), but with the same flow and in the same pressure conditions as those used for the sample gases.

The background signal can be minimized by placing the windows at nodes of the mode being excited and by introducing buffer volumes at both ends of the cell. The ratio of buffer to resonator diameters must be large enough, and the buffer length has to be equal to one-fourth of resonator length according to Dumitras et al. [25]. Harren et al. suggests that the influence of scattered light on the PA background signal can be minimized by use of highly reflecting, polished resonator wall material with a good thermal conductivity, and in the case of infrared light, an acoustic resonator with a polished gold-coated copper wall offers the desired results [67].

The best solutions in reaching the best sensitivity in molecular gas absorption are: amplifying PA signals using resonant cells, optical multipass arrangements, high laser powers, microphone arrays, reduced noise. Sensitivity to detect a particular compound depends heavily on its spectroscopic properties. For example, the absorption lines closely spaced in a Q branch of a strong vibrational transitions helps to achieve low detection limits of such a gas [67].

In the case of the investigation of complex gas mixtures, there are limitations when molecular absorption lines are close to each other. The majority of gases have their absorption bands in the mid-IR spectral region (∼ 2–25 μm) [79]. The most abundant infrared absorbing gases are water and carbon dioxide. Gases can also be separated by gas chromatographic methods, selective trapping inside a cold trap (e.g., water), or by a specific chemical reaction. To prevent the undesired supplementary absorption of the interfering gases, especially carbon dioxide, Bratu et al. used potassium hydroxide (KOH) scrubber (Merck KOH pellets) using four recipients with different volumes (13 cm^3^, 45 cm^3^, 120 cm^3^, and 213 cm^3^, respectively) in a CO_2_LPAS system before the PA cell [80]. They measured the efficiency of the KOH scrubber when it is used for multiple measurements. A quantity of minimum 120 cm^3^ KOH pellets was found to be used for a sampling bag of 750 mL in order to keep the detection of ethylene and ammonia traces free of CO_2_ interference using a CO_2_LPAS technique. Wang et al. reported a new method for the detection of breath ammonia in high concentration of CO_2_ and H_2_O by using a wavelength modulated photoacoustic spectrometer based on a near-infrared tunable erbium-doped fiber laser in combination with an optical fiber amplifier [54]. The multi-wavelength (1522.44 nm, 1522.94 nm and 1545.05 nm) PA signal measurement is established to detect multi-spectrum signal in samples. They solve the problem by detecting ammonia in high concentrations of CO_2_ and H_2_O at atmospheric pressure and obtain a minimum detection limit of 16 ppb (SNR = 1) in simulated breath samples (5.3% CO_2_ and 6.2% H_2_O (100% relative humidity at 37 °C)).

## 3. Applications of PAS in Human Health

### 3.1. Human Breath Biomarkers

In order to obtain accurate information and to determine the concentration of a specific breathing biomarker, it is necessary to have an extremely sensitive technique, but also very selective [7,8,9,11,12,13,14,15,23,24,25,26,67]. Photoacoustic spectroscopy provides a novel approach in the clinic for the analysis of human breath [25,26]. Today, conventional tests for the diagnosis and clinical monitoring focuses on the analysis of blood and urine, causing discomfort and/or embarrassment and require time-consuming assays. In clinical medicine, breath testing is a non-invasive test, causing negligible minimal risk and discomfort for patients [1,2,3,4,5,6,7,8,9,10,11,12,13,14,15,16,17,18,19,20,21,22,23,24,25,26,53,54,67]. Breath odors were used for disease diagnostics long before present-day, patients’ breathing has been characterized by a specific odor from the time of Hippocrates, a sweet smell fruity odor of acetone in patients with uncontrolled diabetes mellitus; the musty, fishy reek of advanced liver disease; the urine-like smell that accompanies failing kidneys; and the putrid stench of a lung abscess [7]. Pauling’s research opened the door to new, non-invasive research, and thus monitoring VOCs in respiration became useful for diagnostic, treatment and therapy monitoring or as well analysis of metabolic gases [11,12,13,81].

In studies on human breath, more than 3000 different VOCs and particles have been detected, where most of the VOCs are at low concentrations, i.e., from ppm to ppb or ppt [81,82,83,84,85,86]. A healthy adult human has a respiratory rate of 12−15 breaths/min at rest, inspiring and expiring 6–8 L of air per minute. The breath air is a mixture of nitrogen (78%), oxygen (16%), carbon dioxide (4–5%), carbon monoxide (0–6 ppm), ammonia (0.5–2 ppm), inert gases, and traces of VOCs (0.9%), water vapor (about 6% at saturation) and with an oral exhalation relative humidity of 82–85% [82].

The most predominant endogenous compounds in breath are isoprene (12–580 ppb), ethanol (13–1000 ppb), methanol (160–2000 ppb), and acetone (1.2–1800 ppb); other alcohols are present in the very low concentration. Only a small number of VOCs are common to everyone (isoprene, acetone, ethane, and methanol) which are products of metabolic processes and some of these VOCs (ethane, *n*-pentane, butane, ethanol, acetone) have been identified as breath biomarkers [81,82,83,84,85,86].

In addition to these VOCs, exhaled nitric oxide, hydrogen, ammonia, and carbon monoxide are related to health condition and can reflect a potential disease of the individual or a recent exposure to a drug or an environmental pollutant [87,88]. 

#### 3.1.1. Ammonia

Ammonia (NH_3_), present in all body fluids, is a key component of the nitrogen cycle which results from protein metabolism and arises naturally from metabolic processes in the stomach and intestines [54]. Ammonia has been linked as a biomarker to liver and kidney function and to the effects of exercise, bacterial activity, and halitosis [22,89,90,91]. When the liver and/or kidneys are not functioning correctly, the blood cannot be filtered properly and this leads to high levels of ammonia or hyperammonaemia. The excess NH_3_ diffuses into the lungs and can be exhaled in the breath. Ammonia molecules are very reactive and hence challenging to measure. LPAS has the potential to be a viable tool for monitoring real-time concentrations of NH_3_ in human breath. The level of NH_3_ in human breath was measured to be between 50 ppb and 2000 ppb, depending on a number of factors, including the patient’s health, the route of sampling (nasal or oral), the contribution of oral bacteria, diet, drug use, physical and metabolic activity [89,90,91]. Hibbard and Killard measured the breath NH_3_ concentrations of a normal healthy population using a LPAS device [91]. They found an average level of breath NH_3_ of 265 ppb, ranging from 29 to 688 ppb with a higher breath ammonia concentration in male volunteers. S.F. Solga et al. measured breath NH_3_ using QEPAS method that uses a quantum cascade-based laser [92]. They found that healthy individuals may begin the day with a breath NH_3_ measurement of 100–1,000 ppb. Lewicki et al. reported also a QCL breath sensor platform for medical applications that employed quartz-enhanced photoacoustic spectroscopy and found a detection sensitivity for exhaled NH_3_ < 10 ppbv level with 1 s time resolution [93].

#### 3.1.2. Nitric Oxide

Breath nitric oxide (NO) is a biomarker for asthma, bronchieactasis, and rhinitis [94,95]. Dong et al. showed an optimized configuration applied to detect NO at 1900.08 cm^−1^ (5.26 µm) free from H_2_O and CO_2_ interference [96]. They present the optimized geometrical parameters of micro-resonator for a QEPAS sensor to perform sensitive and background-free spectroscopic measurements using mid-IR QCL excitation sources and obtained a sensitivity of 4.9 ppbV is achieved with a 1-s averaging time and 66 mW optical excitation power. Gondal et al. developed in their laboratory a highly sensitive sensor based on the PAS principle for detection of NO at very low concentration (ppbV) for applications such as environmental testing and human health [97]. In order to optimize the PAS signal and to obtain a greater sensitivity, they investigated the parametric dependence, in which the dependence of the PAS signal on the NO gas pressure, the cell geometry, the buffer gas (Ar, N_2_, He) and the laser pulse energy using three locally developed PAS cells. Thus, the best PAS signal to noise ratio they acquired by using a cylindrical cell having three acoustic filters and argon as a buffer gas.

#### 3.1.3. Carbon Dioxide

The carbon dioxide (CO_2_) concentration in the breath is correlated as biomarker for *Helicobacter pylori*, liver malfunction and activity of the bacteria in the body. The CO_2_ concentration in the breath indicates the presence of *Helicobacter pylori* in the stomach, as this bacterium digests urea by releasing carbon dioxide and ammonia [98]. Hiibard and Killard also measured oral breath ammonia and oral breath carbon dioxide using a photoacoustic spectroscopy technique. They studied the relationship between oral breath ammonia and oral breath carbon dioxide [99].

#### 3.1.4. Acetone

In patients with diabetes mellitus, the body produces excess amounts of ketones such as acetone because the body uses fats instead of glucose to produce energy, which are then exhaled during respiration. Acetone has been successfully used as a biomarker for diabetes mellitus, especially in type 1 diabetes mellitus [100]. It has been found that quantification of acetone concentration in human breath, using breath analysis techniques, correlates strongly with acetone concentration in the blood and other ketone bodies such as β-hydroxybutyrate. Thus, measurement of acetone from breath gives better diagnostic control of a patient’s diabetic condition, rather than through the use of blood glucose measurements alone [101].

#### 3.1.5. Etylene

Ethylene (C_2_H_4_) from the human breath is an indicator of oxidant stress and can be directly correlated to physiological events in the patients (or biochemical events surrounding lipid peroxidation). Lipid peroxidation (LP) is the free-radical-induced oxidative degradation of polyunsaturated fatty acids, where biomembranes and cells are thereby disrupted, causing cell damage and cell death. In the human body, the fatty acids inside the membrane lipids are mainly linoleic acid and arachidonic acid. The peroxidation of these fatty acids produces two volatile alkanes: ethylene and pentane respectively. Both of them are considered in the literature to be good biomarkers of free radical-induced lipid peroxidation in humans [102].

Biomarkers are chemicals, usually VOCs, which indicate the presence or severity of a disease, evaluating the most effective therapeutic treatments. These chemicals may be introduced into the organism (exogenous) or produced within the body (endogenous). As a result of extensive studies only a few breath markers have been discovered and used in monitoring or diagnosis of disease (see Table 1).

### 3.2. LPAS for Breath Analysis: Clinical Implementation

#### 3.2.1. Oxidative Stress

Oxidative stress (OS) is a phenomenon caused by an imbalance between free radicals and antioxidants in cells and tissues, reduces the ability of a biological system to detoxify this reactive product [103,104]. A free radical is an unstable and highly reactive molecular species with an unpaired electron that can donate or accept an electron from other molecules [105]. Free radicals can cause large chain chemical reactions in your body because they react so easily with other molecules. These reactions are called oxidation and can be beneficial or harmful. Free radicals are generated both endogenous and exogenous sources. The production of exogenous free radicals can be caused by cancer, infection, inflammation, mental stress, aging or excessive physical exercise. Exogenous free radicals appear as a result of exposure to a number of external factors such as air pollution, heavy metals, certain drugs, radiation, cigarette smoke or alcohol [104]. There are many types of free radicals, but the most reactive species and those of most concern in biological systems are derived from oxygen, and are known as reactive oxygen species (ROS) [105]. ROS include ions, free radicals and peroxides and an increase of these species leads to significant damage cell, damage known as OS [106]. Excess free radicals cause oxidative damage to biomolecules such as lipids, proteins or DNA and lead to many chronic diseases (atherosclerosis, cancer, diabetics, rheumatoid arthritis, myocardial infarction, cardiovascular disease, chronic inflammation, stroke and septic shock, aging and other degenerative diseases) [107]. As a result of oxidation of cellular lipids, free radicals produce cellular lesions, a process known as lipid peroxidation [108,109,110,111]. Some of the stable end-products of LP, such as ethane, ethylene, and 1-pentane are well suited for the estimation of cellular damage because these species are excreted in breath within minutes of their formation in tissues. Such quantification of LP is superior to direct measurement of free radicals since the quantification of damage is more relevant for the estimation of adverse effects [111]. Using a CO_2_ laser-based photoacoustic detector, lipid peroxidation was monitored by measuring breath ethylene during cardiac surgery by Cristescu et al. In addition, OS is influenced by external factors such as cigarette smoke, environmental pollutants and exposure to ionizing radiation [112].

#### 3.2.2. LPAS in Subjects with Autism

Autism spectrum disorder (ASD) is a highly heritable, heterogeneous neurodevelopmental disorder characterized by impaired social interaction and communication as well as restricted, repetitive behavior presenting in early childhood [113,114]. There is no curative treatment for ASD, but early intensive behavioral treatment can significantly improve long-term developmental outcomes [115,116,117]. Recent studies showed measurement of markers to understand the complex systems [118,119,120]. The markers include neurotransmitters, hormones and markers of immune function and inflammation [121,122,123,124,125,126,127]. It has been suggested that OS may play a role in etiopathogenesis of ADS, that neurological changes may be due to OS occurring in early brain tissue development and reduced antioxidative barrier [128]. The etymology of this condition remain unclear, but understanding of the potential role of OS in the pathogenesis of autism would be very useful for the prevention or therapy of this condition [129,130,131,132]. The end product of LP, ethylene, have been considered to be a marker of OS [133]. Bratu et al. analyzed the breath ethylene from the young adults with autism using a CO_2_LPAS system [134]. Ethylene concentrations from breath samples were measured in young adults with autism and the results were compared with a healthy controls. Supplements with vitamin B-complex, Tonotil-N, Neuro Optimizer^®^ 60 cps and cod liver oil were administrated for three consecutive months to the subjects with autism involved in their study. They measured breath ethylene using a CO_2_LPAS and compare the level of OS (given by the exhaled ethylene) from young adults with autism with the level of OS from individuals with healthy physiological state. The LPAS system comprises a CO_2_ laser radiation source, a photoacoustic (PA) cell where the gas sample is enclosed and analyzed, a vacuum/gas handling system and a detection unit. The CO_2_ radiation source is a home-built laser, line-tunable and frequency-stabilized, that emits continuous wave radiation with an output power of 2–5 W, tunable between 9.2 and 10.8 µm on 57 different vibrational–rotational lines and its emission spectrum overlaps with the absorption fingerprint of ethylene. The cw, tunable CO_2_ laser beam is chopped, focused by a ZnSe lens, and introduced in the PA cell (the external resonator home-build). The PA cell is made of stainless steel and Teflon to reduce the outgassing problems and consists of an acoustic resonator (pipe), windows, gas inlets and outlets, and four microphones connected in series (sensitivity of 20 mV Pa^−1^ each).The majority of non-invasive studies have reported increased OS in children with autism, this measurements on young adults reported the opposite, a decrease of ethylene concentration. This discrepancy may be due to several factors such as, different measuring technique, exposure to antioxidant supplements, lifestyle and dietary patterns, different stages of the disease.

#### 3.2.3. LPAS in Subjects with Schizophrenia

Schizophrenia (SCZ) is a common psychiatric disorder, marked by gross distortion from reality and disturbances in thinking, feeling, and behavior. People with SCZ may seem like they have lost touch with reality. It has a life-time prevalence of ~1% of the world’s population. It is believed that increased OS may be relevant to the pathophysiology of SCZ, but most of the results regarding this subject are contrasting [135,136,137]. Behavior disorder in the absence of mental health and social problems is best managed with psychological therapies, but the success rate is variable. Some individuals may, therefore, end up being treated with antipsychotic medications along with other approaches [135,136]. Most previous studies in SCZ have been invasive, requiring samples of blood or cerebrospinal fluid or indirect measures of antioxidant enzyme levels have been used [138]. According to Puri et al. there is a positive correlation between levels of ethane (C_2_H_6_) in expired alveolar breath in human subjects and cerebral levels of phosphodiesters, their study being on patients with schizophrenia because they present an increased free radical-mediated damage and cerebral lipid peroxidation [139]. They correlated systemic OS with changes in brain metabolism that define ethane as the end product of omega-3 PUFA oxidation and tested the hypothesis that exhaled ethane is a biomarker of cerebral n-3 polyunsaturated fatty acid peroxidation in humans. The group reasoned that the cerebral source of ethane would be the docosahexaenoic acid component of membrane phospholipids. Popa et al. used a CO_2_LPAS method to assess the exhaled breath compounds in patients with schizophrenia [140]. The group measured breath ethylene and ammonia in patients with SCZ before/after the treatment with Levomepromazine. From the results of this study, the ethylene and ammonia breaths of SCZ patients were identified in higher concentrations when was compared to the control healthy group. The results also reveal that the ethylene levels can be considered as a measure of OS index in SCZ people and the results support the hypothesis of the oxidant/antioxidant ratio of the balance as a key component that may contribute to SCZ pathology.

#### 3.2.4. LPAS in Smokers

According to the World Health Organisation (WHO), the tobacco smoke kills more than 8 million people a year around the world, more than 7 million of those deaths are the result of direct tobacco use while around 1.2 million are the result of non-smokers being exposed to second-hand smoke. Cigarette smoking represents the major risk factor in the development of lung cancer, which is the main cause of cancer deaths in men and women in the world [141]. Bukreeva et al. investigated the impact of smoking on the air exhaled by patients with chronic obstructive pulmonary disease (COPD) and asthma, by using PAS [142]. The exhaled breath absorption spectra from the subjects with COPD and asthma were compared with those from non-smoking healthy individuals. They observed that the spectra of the compounds exhaled air of asthmatics differed from that of both smoking. The spectra of the breath compounds were recorded on 10R and 10P branch of the CO_2_ laser. Their results make it possible to distinguish non-smoking healthy individuals from asthmatics and COPD patients in 94 and 89% of cases, respectively. Giubileo et al. measured small traces of ethylene down to ppb level in the exhled breath of samples collected prior smoking the cigarette and compared with the samples collected after 30 min following the inhalation of cigarette smoke by means of PAS [143]. Popa et al. used a CO_2_LPAS technology to investigate ethylene as breath biomarker from traditional cigarette smokers (T-cigarettes) vs. electronic cigarette smokers (E-cigarettes) [144]. Their results showed that there is a difference in breath ethylene concentration in active smoking with E-cigarettes vs. T-cigarettes.

#### 3.2.5. LPAS in Radiotherapy

Cancer affects one in three of the population [145]. The main type of radiotherapy treatment is external beam radiotherapy in which high energy X-rays are produced using a linear accelerator and directed in a focused beam, from varying angles, onto the target area of the body. The X-rays used for treatment are at energies typically between 4 and 20 MeV, in contrast to the X-rays used for diagnosis, typically at keV energies [146]. Radiotherapy is delivered in ‘fractions’ (treatment sessions over a number of days) to reduce the unwanted effects of large doses of radiation on normal tissue [147,148].The main mechanism behind radiation therapy is believed to be radiation-induced damage to the DNA of the tumor cells, with subsequent cell death occurring after a series of cell cycles. DNA damage occurs directly through ionization or indirectly through generation free radicals [148]. The most reactive and therefore potentially dangerous, oxygen radical is the hydroxyl radical (HO•) [149]. A laser photoacoustic spectroscopy (PAS) sensor for online ethylene monitoring and an adjustable diode laser absorption spectroscopy (TDLAS) sensor for ethane detection, was developed by Puiu et al. the ENEA Frascati Molecular Spectroscopy Laboratory [150]. During the experimental study in cooperation with the Umberto I Hospital (Radiology Institute) of Rome, they were able to detect very low concentrations (under 1 ppb) of trace ethylene content in the air exhaled by patients, following X-ray therapy. Another study in this direction has been carried out by Popa et al., which measured breath ethylene using a CO_2_LPAS [151]. The CO_2_ laser is especially useful for detecting ethylene, because, in the first case, one of its laser lines, 10P(14) transition near 10.53 μm, overlaps with the ethylene’s strongest spectral features. They have monitored the evolution of the oxidative attack before, immediately after and at 15 min from the radiotherapy using the exhaled ethylene as a biomarker and observed that at patients with cancer and particularly at those exposed to radiation treatment, the ethylene level is increased, proving the presence of oxidative attack.

#### 3.2.6. LPAS in Lung Cancer

Lung cancer is one of the leading causes of death worldwide and one of the most popular type of cancer. Traditional methods of diagnosing lung cancer are invasive, expensive and time consuming procedure [152,153]. According to Tainavn et al. some volatile organic compounds could be possible candidates for cancer markers [154]. Breath analysis by determining (VOCs) was used by different groups to distinguish between lung cancer patients and a healthy control group [155,156]. Saalberg et al. developed a sensor based on PAS for six VOCs (2-butanone, 1-propanol, isoprene, ethylbenzene, styrene, and hexanal) linked to lung cancer [157]. As a radiation source, the group used an OPO in a wavelength region from 3.2 µm to 3.5 µm. The detection limit for a single substance of the PA sensor was between 5 ppb and 142 ppb. They found that each lung cancer biomarker shows a very characteristic spectrum in the mid-infrared region. Marcus et al. developed a new optical sensor for VOCs that employs an especially compact and simple set-up based on PAS [158]. Using optical detection they measured *n*-butane (C_4_H_10_) as a biomarker for early-stage bronchial carcinoma (lung cancer) with a detection limit for butane in air in the ppb range. They consider that after optimization the sensor has the potential for early-stage lung cancer diagnostics. Another group that use PAS to detect breath biomarkers in the exhaled breath of patients with bronchopulmonary diseases, including lung cancer (LC) patients (N = 9); patients with chronic obstructive pulmonary disease (COPD) (N = 12); patients with pneumonia (N = 11) and a control group of healthy volunteers was those of Kristenev [159]. Petrus et al. also used a laser photoacoustic spectroscopy system for a quantitative analysis of OS by measuring breath ethylene at subjects with non-small cell lung cancer before and immediately after the chemotherapy and in COPD [160]. This quantification is superior to the direct measurement of free radical because can be estimated the cellular damage and the adverse effects. The LPAS system setup used in the present work consists of a radiation source, a PA cell, a vacuum/gas handling system and as a radiation source they used a CO_2_ laser home-built, line-tunable between 9.2 and 10.8 μm on 73 different vibrational-rotational lines and frequency-stabilized, with an output power of 2–5 W. The subjects with lung cancer presented a low level of breath ethylene concentration comparing with the subjects with COPD, but after the chemotherapy session they presented a very high concentration of ethylene in the exhaled breath.

#### 3.2.7. LPAS in Kidney Failure

Chronic kidney disease (CKD) is a progressive disorder, and patients with end-stage renal failure need treatment by transplantation or dialysis. The dose of dialysis is prescribed on the basis of urea removal measures [161]. The treatment at patients undergoing haemodialysis (HD) is administered at least three times a week for 4–5 h, are people that need four or more sessions per week to keep healthy, and some people are fine with only two sessions per week—this is usually people who are older in order to ensure optimum toxin removal [162]. Toxins that are more relevant to HD are not used as biomarkers due to technical difficulties, although a rapid, low cost measurement method would be desirable. Breath gas analysis could meet the requirements as a non-invasive method to provide valuable information about disease processes or metabolic disorders. The breath biomarkers present in the exhaled breath of patients with CKD can be detected and quantified by sensitive analytical techniques. A special attention is given to biomarkers resulting from dialysis-dependent CKD, especially NH_3_, as a potential estimator of the severity of uremia [163]. In a healthy individual, NH_3_+/NH_4_+ are converted into urea in the liver through cycles of urea and citric acid, and urea is transported through the bloodstream to be excreted by the kidneys in urine. Thus, the high concentrations of NH_3_ in the breath, concentrations that exceed the physiological values are due to renal insufficiency. Kidney failure is one of the diseases identified by extremely high NH_3_ content in human expired breath gas. The NH_3_ odor in the mouth of kidney failure patients is associated with high levels of blood urea nitrogen (BUN) [163,164,165]. Popa et al. measure breath ethylene and ammonia at patients with renal failure undergoing HD using a LPAS system that comprise a CO_2_ laser that emit radiation in 9.2–10.8 µm [166]. C_2_H_4_ was measure at 10P(14) laser line, with a coefficient of 30.4 cm^−1^atm^−1^ and NH_3_ at 9R(30) laser line, with a coefficient of 57 cm^−1^atm^−1^. The group demonstrated that HD determines simultaneously a large increase of the C_2_H_4_ concentration in the exhaled breath (owing to the OS) and a reduction of the NH_3_ concentration, correlated to the level of BUN. Also, Wang et al. developed a PA spectrometer based on near-IR tunable fiber laser used for breath ammonia analysis at patients with end-stage renal disease undergoing HD [167]. The measurements results showed a decrease of the breath ammonia before and after dialysis treatment. Narasimhan et al. determined spectroscopically breath NH_3_ levels in seven patients with end-stage renal disease while they were undergoing HD [168]. They correlate breath measurements with blood samples for BUN and creatinine. The initial levels of breath NH_3_, i.e., at the beginning of dialysis, were found between 1,500 ppb and 2,000 ppb and observed a reduction in breath NH_3_ concentration relatively slow from this point on to the end of dialysis treatment, at which point the levels was between 150 to 200 ppb. For each breath ammonia measurement, taken at 15–30 min intervals during the dialysis, was also sampled the patient’s blood for BUN and creatinine. The breath NH_3_ data were available in real time, whereas the BUN and creatinine data were available generally 24 h later from the laboratory. They found a good correlation between breath ammonia concentration and BUN and creatinine. For one of the patients, the correlation gave an R^2^ of 0.95 for breath ammonia and BUN correlation and an R^2^ of 0.83 for breath ammonia and creatinine correlation. These preliminary data indicate the possibility of using the real-time breath ammonia measurements for determining efficacy and endpoint of HD. Also, Tittel et al. reported a sensor for quantitative measurements of NH_3_ and NO concentrations present in exhaled breath [169]. The NH_3_ concentration measurements were performed with a 2f wavelength modulation quartz enhanced photoacoustic spectroscopy (QEPAS) technique, which is suitable for real time breath measurements at patients with liver and kidney disorders. They used a Hamamatsu air-cooled high heat load (HHL) packaged CW DFB-QCL that operate at 17.5 °C, on NH_3_ absorption line at 967.35 cm^−1^ (λ~10.34 μm) without interference, with ~ 20 mW of optical power, and the sensor includes a reference cell, filled with a 2000 ppmv NH_3_:N_2_ mixture at 130 Torr, which is used to block the absorption line.

#### 3.2.8. LPAS in Diabetes

According to the World Health Organization (WHO), an estimated 422 million adults were living with diabetes in 2014, compared to 108 million in 1980. In 2016, an estimated 1.6 million deaths were directly caused by diabetes, another 2.2 million deaths were attributable to high blood glucose in 2012 [170]. Type 2 diabetes mellitus (T2DM) has reached epidemic proportions with explosive increase in incidence worldwide over the past few decades, particularly in developing countries, in conjunction with increased obesity rates and westernization of lifestyle [170,171]. The major characteristic of T2DM is hyperglycemia, and it is known as non-insulin dependent or maturity diabetes which generally develops after 40 years of age, but is increasingly being seen at younger ages. The causes if T2DM are inadequate insulin secretion and resistance to insulin action. Predicted to become the seventh leading cause of death, diabetes is dangerous because of its complications: cardiovascular diseases, blindness, risk of amputation, kidney failure, etc. The blood glucose concentration in diabetic patients is the key parameter as maintenance at an appropriate level allows the postponement of these complications [172,173]. The microscopic structure and secretion products of skin tissues had a significant impact on non-invasive glucose measurements in mid-infrared PAS according to the study of Sim [174]. They presented a method based on mid-infrared PAS for non-invasive glucose monitoring based on microscopic spatial information of skin and obtained microscopic spatial information of the skin prior to the spectroscopic measurement using the same laser used for the spectroscopy. Non-invasive methods for blood glucose monitoring preferred or for monitoring the disease complications. In recent years, the technique of PAS has been demonstrated for non-invasive glucose detection due to the higher sensitivity and selectivity [175,176]. Tyas et al. measured breath acetone on subjects with T2DM and a group of healthy volunteers, using a CO_2_ laser PA spectrometer [177]. In this research, the highest observed intracavity power was (49.96 ± 0.02) W for active medium gas composition He: N_2_: CO_2_ at 30:50:50 and the acetone concentrations was measured on 10P20 laser line, and the lowest detection limit set at (30 ± 4) ppb. Using a CO_2_LPAS techniques, Petrus et al. measured breath C_2_H_4_ in subjects with T2DM [178]. T2DM subjects underwent both breath analysis and blood tests to determine glycated hemoglobin A1c(HbA1c). Breath C_2_H_4_ was found in high concentration in diabetics compared to healthy subjects and a much higher concentration in subjects with T2DM with complications. Through this study, C_2_H_4_ breathing can be considered an indicator of OS and poor glycemic control in T2DM subjects. The same research group, measured breath NH_3_ concentrations in subjects with T2DM using a CO_2_LPAS [179]. The NH_3_ concentration of healthy subjects was in the range of 0.832 ppm and 1.76 ppm, but at the subjects with T2DM the NH_3_ concentration range was between 2.74 ppm and 10.16 ppm. They observe a higher level of ammonia in the breath of diabetics subjects compared to healthy persons, but also an increase in ammonia concentration in diabetic subjects that present hypertension and/or inflammatory syndrome. A novel approach with a QCL-based photoacoustic sensor toward non-invasive glucose monitoring through human skin was presented by Sigrist [180]. The group reported the first steps to target non-invasive glucose monitoring by developing a new scheme based on midinfrared LPAS. They set the QCL to the 1034 cm^−1^ glucose absorption peak for various glucose concentrations to determine the limit of detection (LOD). In this way they achieved an LOD of 30 mg/dL (for SNR = 1) for aqueous glucose solutions, and an LOD of 50 mg/dL (SNR = 1) for keratinocyte solutions.

#### 3.2.9. LPAS in Surgical Smoke Gases Detection

Surgical smoke results from tissue interaction with surgical instruments such as lasers, high-frequency electric knives, and ultrasonic/harmonic scalpel [181]. Surgical smoke consists of 95% water and 5% cellular debris in the form of particles such as chemicals, blood and tissue particles, viruses, and bacteria [182]. Surgical smoke has been shown to exhibit potential risks for surgeons, nurses, anesthesiologists, and technicians in the operation room due to long term exposure of smoke [183]. The chemicals are gases and vapors and consist contains more than 80 different toxic chemicals and byproducts such as: acrolein (a known carcinogen), acetonitrile, acrylonitrile (long term exposure causes cancer), benzene (a known carcinogen), butadiene (a known carcinogen), carbon monoxide, ethylene, formaldehyde (a known carcinogen, used to preserve surgical specimens and as an embalming fluid), free radicals, hydrogen cyanide (neurotoxin used in chemical warfare, is cardio-toxic), ammonia, toluene (a known carcinogen), etc. [181]. Ray et al. reported a quantitative model study where they first used laser CO_2_LPAS and focused on analyzing VOCs produced thermal and radiofrequency bipolar cautery on porcine liver [184]. They detected carbon dioxide, water vapor, ammonia, ethanol and methanol in the ppm to sub-ppm range and molar fractions, and the concentrations of methanol and ethanol differed between the two cautery devices employed. Petrus et al. found traces of benzene, ammonia, ethylene and methanol in surgical smoke in the range of ppm and acrolein and acetonitrile in the range of ppb, in an environment that is mostly composed of water and carbon dioxide [185]. They investigated the relationship between the gas concentrations and laser power and exposure time. Sigrist reported the first in vivo study on surgical smoke performed at the University Hospital in Zurich, where experiments were realized using laser spectroscopy and the smoke samples were collected in Tedlar bags during the course of several operations [180]. In addition to water vapor traces of methane, ethane and ethylene and higher concentrations of anesthetic gas sevoflurane, over 450 ppm have been detected. Hubner reported also the measurements carried out on samples of smoke produced during laparoscopic surgery of the colon using a bipolar vessel sealing device (LigaSure™) [186]. Samples were analyzed for CO_2_LPAS and confirmed by a Fourier transform infrared spectrum. They report that the absorption spectra differ considerably between patients and detected a broad absorption line at 100 ppm which indicates H_2_O and more unknown molecules, but no known toxic substances such as phenol or indole have been identified.

#### 3.2.10. LPAS in the Study of Dietary Effects on VOCs

Various volatile compounds can be present in human breath depending on nutrition, metabolic state including diseases and medication, microbial infections and personal oral hygiene [187,188]. Volatile metabolites in exhaled human breath come from several sources such as: the inspired air, the microorganisms in the mouth and/or nose, system lung, gastrointestinal tract, and human metabolism [189]. The effects of these factors may either be due to a direct impact on metabolism, or because they alter the gastrointestinal flora, bacteria being a major source of compounds in the breath [190]. In other words, the effects of dietary constituents on breath composition are complex and detailed information about the impact of food consumption on breath composition is lacking. The VOCs emitted in the exhaled breath after garlic ingestion was assessed by PTR-MS was reported by Taucher et al. and the results showed a variation in VOCs levels in time [191]. Also, according to Saha et al. overnutrition may generate free radicals, and subsequently elevate OS, in other words, lifestyle plays an important role in the development of OS that can cause changes in the DNA [192]. Petrus et al. monitored the response of the organism to different food habits (mixed, vegetarian, raw vegan and Dukan) using a CO_2_LPAS technique [193]. Exhaled breath was collected from healthy, non-smoking volunteers with different food diets, different physical activity, no food supplements or pillows, and with a mean body index (BMI) of 22 ± 2.7. They measured breath C_2_H_4_ and breath NH_3_ concentrations and found that the C_2_H_4_ and the NH_3_ concentrations differ by adopted diet and also differ from one person to another.

## 4. Conclusions

This review presents an update of the current status of photoacoustic spectroscopy technique for clinical breath gas analysis and describes their applications. Photoacoustic spectroscopy techniques offer unique possibilities for monitoring chemical species from multi-component gas samples in medical applications.

Laser-based photoacoustic detectors are able to monitor trace gas concentrations under atmospheric conditions with high sensitivity within a small volume of gas, non-invasively and on-line under dynamic conditions. Numerous PAS methods have been developed during several decades and their potential in the medical clinic has been recognized by the numerous applications. The applications of PAS in the clinic include cancer therapy, renal failure, diabetes mellitus, autism, schizophrenia and more. 

The detection of VOCs from the exhaled breath represents an attractive non-invasive tool for monitoring and diagnosing disease. Researchers are seeking explanations for the observed breath biomarker-disease correlations, but more research is needed for further development in this field.

## Figures and Tables

**Table 1:** Breath markers in disease.

Breath Biomarker	Diseases	References
Ethane, Ethylene	Oxidative stress: Lipid peroxidation	[102]
Nitric oxide, Carbon monoxide, H_2_O_2_, isoprostanes, nitrite/nitrate	Lung disease: Asthma, COPD, lung cancer	[94,95]
Acetone	Metabolic disorders: Diabetes	[100,101]
H_2_, Carbon dioxide	Gastroenteric diseases: gastritis, ulcer, Helicobacter Pylori	[98,99]
Ammonia	Liver and/or kidneys disease	[54,89,90,91,92,93]
